# Cost-Effective Train Presence Detection and Alerting Using Resource-Constrained Devices

**DOI:** 10.3390/s25196045

**Published:** 2025-10-01

**Authors:** Dimitrios Zorbas, Maral Baizhuminova, Dnislam Urazayev, Aida Eduard, Gulim Nurgazina, Nursultan Atymtay, Marko Ristin

**Affiliations:** School of Engineering & Digital Sciences, Nazarbayev University, Astana 010000, Kazakhstan; dimitrios.zorbas@nu.edu.kz (D.Z.); maral.baizhuminova@alumni.nu.edu.kz (M.B.); aida.eduard@alumni.nu.edu.kz (A.E.); gulim.nurgazina@alumni.nu.edu.kz (G.N.); nursultan.atymtay@nu.edu.kz (N.A.); rist@zhaw.ch (M.R.)

**Keywords:** railways, Machine Learning, edge computing, LoRa, low-power

## Abstract

Early train detection is vital for ensuring the safety of railway personnel, particularly in remote locations where fixed signaling infrastructure is unavailable. Unlike many existing solutions that rely on high-power, high-cost sensors and compute platforms, this work presents a lightweight, low-cost, and portable framework designed to run entirely on resource-constrained microcontrollers with just kilobytes of Random Access Memory (RAM). The proposed system uses vibration data from low-cost accelerometers and employs a simple yet effective Linear Regression (LR) model for almost real-time prediction of train arrival times. To ensure feasibility on low-end hardware, a parallel-processing framework is introduced, enabling continuous data collection, Machine Learning (ML) inference, and wireless communication with strict timing and energy constraints. The decision-making process, including data preprocessing and ML prediction, completes in under 10 ms, and alerts are transmitted via LoRa, enabling kilometer-range communication. Field tests on active railway lines confirm that the system detects approaching trains 15 s in advance with no false negatives and a small number of explainable false positives. Power characterization demonstrates that the system can operate for more than 6 days on a 10 Ah battery, with potential for months of operation using wake-on-vibration modes.

## 1. Introduction

Early detection of trains is a critical aspect of ensuring safety and efficiency in the transportation sector. Detecting trains in advance allows for timely signaling, scheduling, and coordination of activities on the railway that are essential for preventing accidents, such as serious injuries and deaths. According to the National Safety Council, there were 3316 injuries and 10 deaths among rail employees on duty in 2022 in the United States (https://injuryfacts.nsc.org/home-and-community/safety-topics/railroad-deaths-and-injuries/ (accessed on 29 August 2025)). The Ministry of Railways in India stated in 2023 that 361 railway workers died in the past five years in India due to train accidents (https://www.thehindu.com/news/national/parliamentary-proceedings-361-railway-workers-died-on-duty-over-the-past-five-years-railway-ministry-tells-rajya-sabha/article67627626.ece (accessed on 29 August 2025)).

A number of solutions have recently been proposed in the literature to analyze data from various sensors and sources to accurately predict arrivals of trains. For example, accelerometers can be employed to measure the intensity of vibrations on rails and detect the train’s presence even if the latter is located hundreds of meters away [1]. By analyzing accelerometer data, early detection systems can estimate when a train is approaching a particular section of the track, allowing for timely warnings to be issued to other trains or personnel working on the track [2]. However, such train arrival detection systems frequently necessitate substantial processing power to ensure responsiveness suitable for time-critical scenarios.

To prevent accidents, a train detection system must meet several critical requirements and address key design challenges:

1. Timely Detection and Notification: The system must execute the tasks of perception, analysis, and signaling within a few seconds. Additionally, the total time between train detection and its arrival at the work site must be no less than 15 s to allow sufficient time for personnel to evacuate the tracks. This time requirement is defined by the railway company and represents the maximum interval needed for personnel working on or near the tracks to safely clear equipment or move away from the railway line.
2. Affordability and Cost-Effectiveness: The overall cost of the system must be low enough to ensure accessibility and adoption across both developed and developing regions. This affordability is essential for enabling widespread deployment and improving railway safety globally, regardless of budget constraints.
3. Portability and Infrastructure Independence: Given that many remote regions lack permanently installed safety systems, the proposed solution must be compact and portable. It should be capable of operating autonomously for several hours—or ideally days—without requiring maintenance or fixed infrastructure support.
4. Efficient and Reliable ML Inference: The choice of ML model introduces further challenges. The model must (i) reliably distinguish between actual train-induced vibrations and irrelevant noise (e.g., hammering on the rail), (ii) be compact enough to run on resource-constrained embedded devices, and (iii) perform inference within strict timing constraints to support real-time decision-making. The inference time must be much shorter than the duration of the data collection window, giving enough time to other operations such as data pre-processing and decision transmission.

**Figure 1:**
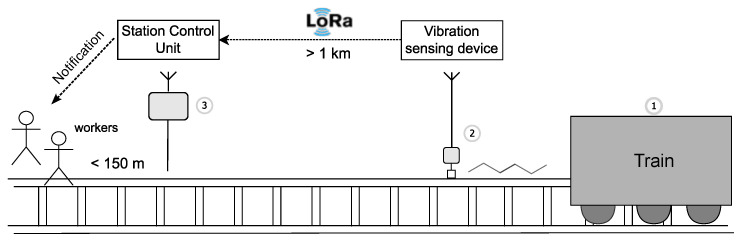
Overview of the examined scenario: ➀ the train arrival time is estimated using a vibration sensing device; ➁ a notification packet is generated and transmitted over LoRa; ➂ the packet is received by a station control unit and forwarded to personnel to notify them to evacuate the rails.

In lieu of this, we propose a lightweight framework for a near-real-time ML-powered prediction system that can be deployed on resource-constrained devices, as illustrated in Figure 1. The system is intended to be deployed in remote areas, far from cities or stations, where no permanent signaling infrastructure exists. This means that it must meet the requirements described earlier; to be portable, low-cost, and last as long as the repair work is going on. There is no restriction in the type of train to be detected. The system operates by alerting the approach of the train via reading the vibration data from the rails, predicting the arrival time of the train by utilizing an ML model, and propagating the alert to the railroad personnel via a custom LoRa-based ad-hoc network. In this paper, we mainly focus on how the train arrival time can be estimated by the deployed vibration sensing device, which consists of a low-cost and low-power microcontroller unit (MCU). After evaluating several ML approaches for their feasibility to be deployed on resource-constrained devices and their feasibility to accurately predict train arrivals, we conclude that a simple LR model is capable of providing reliable results with high accuracy. Next, we assess the feasibility of the proposed scheme using real-world experiments. Specifically, we evaluate whether the system is capable of detecting vibration activity, running the ML decision process for train arrival prediction, and reporting the results using the LoRa radio communication technology. The experiments are conducted using off-the-shelf ESP32 microcontrollers and sensors, soldered together on circuit boards, exhibiting low cost, high accuracy, and long lifetime using commonly used batteries.

The contributions of this work are summarized as follows:

- A cost-effective and low-maintenance system for predicting the arrival of railway trains based on LR is proposed. It retains high accuracy and low false-positive detection. It encompasses simultaneous data collection, data processing, ML inference, and LoRa data transmissions.
- Many popular ML algorithms are also evaluated within the proposed framework to detect the presence of trains using accelerometer data.
- Two prototype versions are developed using off-the-shelf resource-constrained devices. The feasibility of the proposed approach is shown in real-world experiments conducted by the current framework operator, the Kazakhstan National Railways company, Qazaqstan Temir Joly.
- A power characterization of the proposed systems is conducted, which reveals a several-day lifetime using commonly used power supplies, which is more than satisfactory for ad hoc system deployments.

The rest of the paper is organized as follows: Section 2 goes through several train detection approaches in the literature and categorizes them according to the technology they use to detect trains. Section 3 presents an overview of the proposed framework and its components. Section 4 describes the feature engineering and ML model training, while Section 5 presents the ML evaluation using several methods and assessing their performance on resource-limited MCUs in the acquired dataset. Section 6 presents the experimental setup, insights of the system implementations, as well as evaluation results of the experimentation and takeaways. Finally, Section 7 draws conclusions and future research directions.

## 2. Related Research

There are several train detection methods and technologies in the literature, many of them employing or combining different sensing solutions. Techniques such as video, radar, and acoustic sensing were extensively analyzed during a prior decade. Nonetheless, the emergence of ML-driven methods, including deep learning-based obstacle detection in metro systems [3] and extensive deep learning-based railway monitoring assessments [4], has led to the obsolescence of numerous conventional techniques, which have been overshadowed by more precise and versatile intelligence-driven alternatives.

Some of the techniques that offer robust operation are utilized in the industry. A comparison of various technologies for train detection, from research and industry, is shown in Table 1. Most of the industry solutions offer a binary on-the-spot detection method for trains, without providing a solid prediction of the train arrival time. Thus, the purpose of this section is to give an overview of recent approaches in train detection, emphasizing ML approaches, and to categorize them according to the sensing technology they use.

### 2.1. Visual Systems

The employment of various types of visual-based devices has long been integral to transport-related endeavors. Visual scrutiny of railways and roads through computer vision is extensively employed for diverse purposes, such as pedestrian detection on tracks [13,14], vehicular detection [15], obstruction detection [14], junction traffic light classification [16], and background subtraction-based object detection techniques [17]. All of these approaches can be adapted for railroad scenarios by making minor alterations. Visual systems have the potential to serve as a train detection solution in areas with stringent success rate requirements, such as densely populated regions and boarding platforms. However, their feasibility is constrained by the high power consumption and bandwidth requirements of the computer vision models, rendering them unsuitable in remote areas. A typical on-the-edge computer vision setup is based around Raspberry Pi devices, which consume 4–5 W of power. Moreover, the system has to be constantly active and is affected by the low visibility conditions, which may happen due to poor weather conditions. Running computer vision models that are optimized for on-the-edge applications have an additional overhead of over 1 W to achieve results acceptable for an emergency detection system [18]. Finally, as these systems employ more powerful equipment, their financial cost is also higher.

### 2.2. Radar Technologies

Radar-based sensors, such as Light Detection and Ranging sensor (LiDAR), were also adopted for automatic train detection. Pure LiDAR-based roadside sensing solutions, such as those reported by Lin et al. [19] and Jin et al. [20], achieve detection ranges of at most ∼100 m, even with ML/DL (Deep Learning) enhancements. At 50 km/h (13.9 m·s^−1^), a vehicle traverses 100 m in only ∼7.2 s, providing a warning horizon that is generally insufficient for personnel-evacuation scenarios. These radar technologies are often integrated into multi-sensor networks to improve arrival estimation and address the limitations of individual sensor systems. For instance, Zhangyu et al. [21] combine LiDAR with traditional cameras for enhanced accuracy, as LiDAR adds depth perception to video data. Dias et al. [8] developed a combo LiDAR–GNSS (Global Navigation Satellite System) system that precisely detects train arrivals at an up to 500 m range. While using advanced radar technologies offers high performance, it entails high costs and ongoing intensive maintenance.

### 2.3. Acoustic Sensing

The usage of microphones as a means of collecting acoustic data for further classification is a simple solution that does not require complex installation procedures. Researchers employed various methods to detect the presence of an approaching vehicle near the microphone(s) sensing location. For example, Sato et al. [22] used an ML approach to classify the data from two microphones installed in a single spot near the railway. Similarly, data from two microphones that are mutually spaced can also be compared to extract more data from the sound. This setup allows one to determine the speed and direction of the trains based on the difference in sound profiles of two microphones [23].

Acoustic data collected by utilizing Distributed Acoustic Sensors (DAS), which are installed across the railroad, has the potential to be used for high-precision train detection. While the application of DAS undoubtedly achieves both high accuracy and robustness, such installations do, however, incur significant costs in terms of infrastructure, data processing, and labor [24,25].

### 2.4. Vibration Sensing

Vibration signal analysis has emerged as a valuable tool for monitoring machines and mechanical equipment conditions, diagnosing faults, identifying activities, and classifying events [1,26,27,28,29]. Analyzing train track vibrations is crucial for ensuring vehicle effectiveness, passenger comfort, and public safety. Vibration analysis is not computationally costly, which means that vibration sensing systems can leverage real-time data processing and edge computing. Furthermore, vibration sensors are relatively easier to install, as their deployment typically involves only ensuring physical contact with the target structure. Therefore, accelerometer-based train detection systems, highlighted in various studies, offer the potential for early train detection using simple hardware [1]. The main drawback of vibration sensing is defined in the literature as the high sensitivity to surrounding noise [2]. However, these systems can be highly effective when integrated with ML, as they can classify train approach events by identifying specific vibration patterns [1]. Other events could be noise detected from the neighboring tracks, resulting in a false-positive prediction. This could be addressed by pre-processing the data before prediction by using the Fast Fourier Transform (FFT) and Sliding FFT [30]. This approach is, however, too slow for a real-time application on the edge, as is demonstrated in Section 6.2.

### 2.5. Train Prediction on Resource-Constrained Devices

While previous systems effectively leveraged data for vehicle or environmental predictions, the existing literature lacks a specific framework suitable for low-power, resource-constrained edge devices. Notably, Abdel Magid et al. [31] and Elliott et al. [32] achieved successful on-the-edge classification on devices such as the Raspberry Pi 3, BeagleBone Black, and Samsung Galaxy S9. Despite their compact size, these devices are still deemed to possess high computing power within the context of Internet of Things (IoT) standards, as they are capable of running full-fledged Linux-based operating systems. Der Yang et al. [33] showcased a real-time on-the-edge classification example, utilizing a drone to classify images of fields. However, it is noteworthy that the data for inference in this scenario was offloaded to a server. This approach proves impractical in environments without network infrastructure where only low-power IoT devices are available.

Table 1 provides a feature comparison between the proposed system and other approaches in the literature or commercial solutions. We can observe that all current industry solutions use sensors to detect the presence of a train; however, there is no time arrival estimation. This practically means that the train must first arrive at the place of the measurement to be detected, limiting the alerting time. Although communication technologies can transmit alerts within seconds, relying solely on train detection at the measurement point may not guarantee the required early warning time, especially if the communication range is limited due to the terrain characteristics or if environmental conditions degrade the signal quality. In such cases, the train may be too close to the work site by the time the alert is issued, leaving insufficient time for safe evacuation. Therefore, in-advance prediction of train arrival is essential to extend the warning horizon and ensure that alerts are triggered early enough to consistently meet the 15-s safety margin, even under constrained communication conditions.

Furthermore, existing approaches in the literature either require permanent infrastructure or are based on visual detection, which is power-hungry and requires visual contact of the train. The approach proposed in this paper fills the gap between accuracy, time estimation, low cost, and energy efficiency. Moreover, running models on resource-constrained devices is essential for real-time train prediction using accelerometer data, as they enable the prediction of train approach events with minimal hardware requirements and cost. Besides low cost, by processing accelerometer data locally, these systems can predict train approach events with minimal energy consumption, ensuring both accuracy and extended service time in operation.

## 3. Proposed Framework

This section presents the overview of the proposed framework for data collection, preprocessing, prediction, and communication for low-power IoT devices. The proposed framework uses a discrete window approach, which is explained in the next paragraphs.

Task execution on edge devices can be performed either sequentially or in parallel. High-end IoT devices have sufficient processing power to achieve high performance through concurrent execution. However, for low-end microcontrollers, parallel execution is often preferable, as it helps improve their limited processing capabilities.

**Figure 2:**
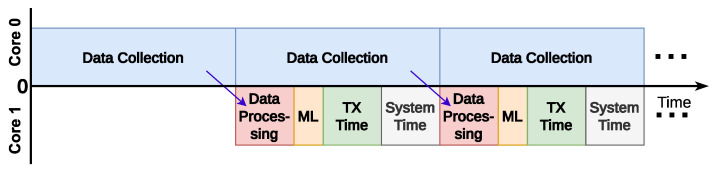
Data capture, processing, ML prediction, and transmission run in parallel. The system continuously estimates train arrival time, and if it falls below a threshold, an alarm is sent to the base station. (Processing is illustrated longer than in practice; transmission time depends on the radio technology).

To achieve parallelism and take decisions as quickly as possible, several tasks are split between the MCU cores; one core is devoted to the continuous data collection process and the other core is responsible for processing data, executing the prediction, and transmitting a packet if a train is detected. The visual representation of the framework is presented in Figure 2.

The parallel execution of data collection and processing presents a dilemma on how to achieve efficient performance on resource-constrained MCUs. There are two main approaches to handle the problem of windowed real-time processing. The most common one is the *sliding window*. The second one is the *discrete window* or the tumbling window processing, in which there is no overlapping between two successive windows. In both cases, data loss during capture cannot be tolerated. One could highlight that the second approach is not purely real-time, in which the computation is done on every data instance, but is close to real-time processing.However, the discrete window approach was chosen over the sliding window approach because (a) the latter causes significantly higher computation cost—due to the continuously overlapping data periods—which cannot be handled by a low-end IoT device; (b) the latter one causes numerous repeated model predictions for similar data requiring additional programming effort to fine-tune the sliding-window length depending on the nature of data; (c) the continuous prediction does not leave much space for transmissions.

The framework facilitates the execution of the prediction model, with the primary stipulation being that the prediction and data transmission processes are considerably shorter than one window size. The window size is dictated by the duration of the data collection process on the main core. Concurrently, the time requirements of the prediction process are contingent upon the volume of input data points, which in turn depends on the frequency of data readings from the main core. However, it is imperative to avoid overly frequent data readings, as this may result in violation of deadlines due to the longer prediction time. Thus, both the data collection window and the prediction process must strike a delicate balance between the quantity of data points, reading frequency, and prediction speed. Moreover, the speed of prediction is contingent upon the selected approach, necessitating the testing of various other approaches on resource-constrained devices. Beyond temporal constraints, the accuracy of prediction must also meet a requisite threshold to ensure usability in critical scenarios.

## 4. Prediction of Train Arrival on the Edge: Feature Engineering and Model Training

**Figure 3:**
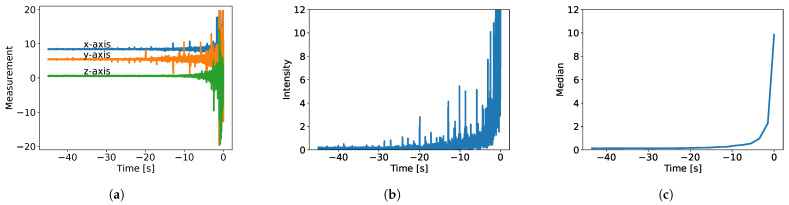
Feature engineering depicted on a train arrival: (**a**) The signal, as recorded with the accelerometer; (**b**) rotation-invariant intensity of the signal; (**c**) medians of the discrete windows.

The purpose of this section is to propose a model capable of running within the time limits of the proposed framework on resource-constrained edge devices, and estimate train arrivals at the point of measurement at least 10 to 15 s in advance. The input to our prediction model consists, by design, of accelerometer measurements spanning in the x-, y-, and z-axis, as depicted in Figure 3a. All axes are relative to the accelerometer positioning. We note that the increase in vibration intensity as the train approaches reflects the well-established physical principle that proximity to the vibration source amplifies the transmitted energy at the sensor location [34].

The data is collected over a predefined window. Based on this window, the model estimates the train’s time-to-arrival (TTA). When the predicted TTA falls below a threshold, e.g., 15 s, an alarm is triggered and transmitted over the network.

### 4.1. Feature Engineering

The accelerometer data in the time domain is too volatile and fine-grained to serve as a reliable model input (see Figure 3a). Minor rotations of the accelerometer or transient noise spikes (e.g., stones or tools hitting the rail) can significantly distort the signal. In theory, frequency-domain analysis could identify actual trends while filtering out noise by emphasizing lower frequencies. However, in our setting, this approach is impractical due to the high computational cost of the frequency transformation (see Section 6.2). Instead, we propose a faster and simpler transformation. First, to ensure rotation invariance, we use the norm of each data point rather than individual axis values (see Equation (1)):

(1) $$s=\sqrt{x^{2}+y^{2}+z^{2}},\;$$

where *s* is the resulting intensity; and *x*, *y*, and *z* are the accelerometer measurements along each axis. Second, to suppress high-frequency noise, such as sudden rail impacts, we apply a median filter over the recorded discrete window *w* (see Equation (2)):

(2) $$\begin{array}{cc} \; & m=\mathrm{median}(\{s|s\in w\}),\;s.t.\\ \; & \left|\left\{s|s\in w,s<m\right\}\right|=\left|\left\{s|s\in w,s\geq m\right\}\right|=\frac{|w|-1}{2}. \end{array}$$

**Figure 4:**
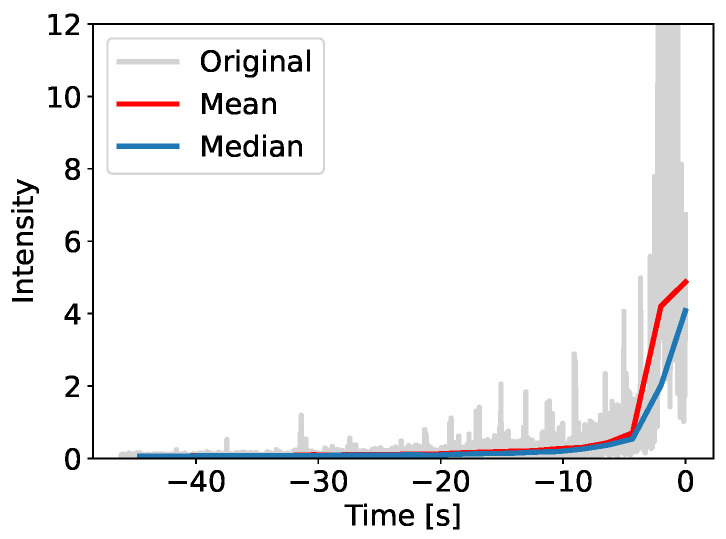
Comparison between mean and median filtering on a train arrival with outliers; the mean is more affected, while median preserves the trend.

Figure 3 depicts our feature transformation. We choose the median filter over alternatives such as the mean filters due to its robustness against outliers. For example, sudden intensity spikes would skew the mean, whereas the median preserves the underlying trend (see Figure 4).

Computing the median requires O(|w|log|w|) operations, which are asymptotically equivalent to frequency transformations such as the FFT. However, in practice, its runtime constant is significantly smaller, as demonstrated in Section 6.2.

One challenge is selecting the window size |w|, as there is a trade-off between small and large windows. A larger window smooths out noise and emphasizes slow trends but introduces delays, since more data must be collected and processed. Conversely, smaller windows enable more frequent predictions but are more susceptible to noise, as they may not capture enough data to preserve the signal. We note that optimizing the sampling-window size requires access to the ground truth, which is not available on edge devices during real-time operation. For this reason, an automatic optimization procedure cannot be executed locally. Instead, the sampling window has to be adjusted offline during the training and validation phases, using annotated datasets, to identify configurations that balance detection accuracy and computational cost. Once determined, these parameters can be deployed on the edge devices for real-time inference.

### 4.2. Prediction Model

To predict TTA, we use a simple physical model, assuming that the TTA is inversely proportional to the signal intensity (see Equation (3)):

(3) $$f\left(m\right)=\frac{a}{m}+b,$$

where f(m) represents the predicted TTA in seconds, *m* is the median intensity over a discrete window, and *a* and *b* are model parameters. These parameters are estimated from a recorded training dataset by minimizing the least squares error. Notably, our model can be rewritten as a standard LR by transforming its input intensity using the reciprocal (see Equation (4)):

(4) $$f\left(m\right)=a\cdot \frac{1}{m}+b,$$

allowing for optimization with any off-the-shelf linear algebra library.

Our model is lightning-fast to compute, requiring only a division and an addition, yet achieves satisfactory performance despite its simplicity, as shown in Section 5. Since the parameters are optimized to the training data corresponding to a specific railroad, the method generalizes well, accommodating variations in intensity characteristics due to different accelerometers, train types, and rail materials.

## 5. Model Evaluation on the Dataset

In this section, we evaluate the proposed memory-frugal model on a dataset recorded in a real-world setting. We compare its performance against other more complex and parameter-rich models. Both the dataset and the code of all the experiments will be available online.

### 5.1. Dataset

The dataset was collected from operational railroad tracks during summer 2024. The material of the track was a hot-rolled steel alloy, which is the most commonly used railway material for high-speed trains in the world. It is worth noting that if the material changes, a new dataset has to be captured, and then the ML model has to be trained on the new dataset to maintain the same level of TTA accuracy. The MPU-6050 accelerometer was used, mounted on a ESP32 microcontroller with 380 KB of RAM and 240 MHz of CPU frequency. Recordings were performed at 88 Hz over 19 h. A total of ten trains passed over the accelerometer during the recording period. We do not differentiate between fast and slow trains, or between passenger and freight trains. Due to the sparsity of train events, most of the data consists of redundant silence. Despite the fact that a highway exists next to the railroad and the presence of rain for a couple of hours during the data capture, the level of noise is extremely low. In fact, the activity caused by a train is hundreds of times higher than any other acceleration recorded in the dataset. This makes the train annotation for model training an easy task. To refine model training and evaluation, we manually annotated train arrivals and segmented the recording into 40 s sessions preceding each train arrival. The first six sessions were used for training, the seventh was held out for validation, and the last three were reserved for testing.

To the best of our knowledge, there is only one similar dataset based on accelerometers published in the literature: “Trainspotting” [1]. The authors focused on classifying trains rather than predicting their arrival, so the sensors were rather insensitive, such that incoming trains were imperceptible in the signal. This made the dataset unsuitable for our application.

### 5.2. Model Training, Learning Objective, and Evaluation Metric

The models were trained to predict TTA for each individual window and evaluated against the actual observed TTA.

Models were trained on the pre-processed vibration data from arriving trains, where data was divided into equal size windows with a sliding-window approach and applying median filtering on the windows. The time remaining until the train hits the sensor was used as labels during training.

Following our framework’s procedure, the model’s performance was assessed based on the TTA error at the moment its prediction crossed a predefined threshold. To align with real-world requirements communicated by engineers of the railway company, we set this threshold to 15 s. For example, if the model predicts a TTA of 15 s when the actual train arrival is 17 s away, the resulting error is 2 s. For fair comparison, all models are given as input the reciprocal of the median window intensity.

Unless stated otherwise, all subsequent experiments use a window size |w| of 2 s, i.e., 176 sample points. The selection and the impact of the window size on model performance is analyzed separately in Section 5.4.

### 5.3. Comparison to Other Models

Our goal is to design the simplest possible model to meet the stringent constraints of edge computing. Thus, it is essential to evaluate the extent, if any, to which our model under-performs due to its simplicity and whether further refinement is necessary. The proposed simple model is experimentally compared to the following two parameter-rich, complex models to assess its effectiveness:

**XGBoost (Extreme Gradient Boosting)** is a fast, regularized implementation of gradient-boosted decision trees, known for strong performance and widespread use in ML competitions. XGBoost is included to assess how much the performance of a non-linear predictor differs from the proposed linear model.

**Long Short-Term Memory (LSTM)** networks are recurrent neural networks designed to capture long-range dependencies using memory cells and gating mechanisms. LSTMs are widely applied in time-series forecasting, including train arrival prediction [35]. Comparing the proposed model to LSTMs allows for an evaluation of the importance of longer-term dependencies in the signal and a direct assessment of a promising alternative approach from the literature.

The hyperparameters of all the models were determined using a grid search based on the performance measured on the validation set. We used scikit-learn 1.6.1, a widely popular Python 3.13 library for ML, to train and evaluate the models.

**Figure 5:**
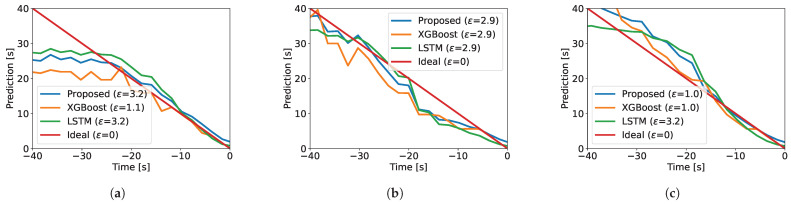
Predicted train arrival times for (**a**) the 8th, (**b**) the 9th, and (**c**) the 10th session. The “ideal” line is the theoretical predictor. The error ε is the deviation from the actual TTA when a model predicts 15 s. All models show similar performance.

Figure 5 illustrates TTA predictions across models over discrete windows preceding train arrivals in the test sessions. A visual comparison reveals that all models perform similarly, with no significant differences. This is also confirmed by comparing the errors between the actual TTA and the predicted 15 s TTA. These findings confirm that our simple model, despite its minimal design, matches the performance of more complex models. Notably, it compares favorably to LSTM—an alternative model successfully applied in the literature [35]—while using orders-of-magnitude-fewer parameters (i.e., only two). The results further indicate that accounting for non-linearity (XGBoost) and temporal dependencies (LSTM) provides limited benefit in our setting.

### 5.4. Effect of the Window Size

We tweaked the window size |w| to estimate how much it impacts the performance of the model. We tested for windows of 22, 44, 88, 176, and 264 samples, corresponding to 0.25, 0.5, 1, 2, and 3 s, respectively. The results, corresponding to Figure 5, are summarized in Table 2. The best performance is achieved with |w|=176, i.e., with windows of 2 s, resulting in the error range 1.0–3.2 s. Hence, this window size balances well the noise suppression while retaining a reasonable prediction frequency in our particular setting. In practice, for novel deployments, the window size needs to be fine-tuned to the specific signal characteristics of the site.

### 5.5. Extended Evaluation in a Realistic Scenario

Apart from evaluating our model’s performance on imminent train arrivals, we also assess its behavior over an extended period, simulating real-world deployments. We run our procedure on all signal recorded five minutes after the seventh validation session. The offset ensures that the train has definitely passed, and that only three remaining train arrivals from the test set are observed. This amounts to 4 h of recorded signal. Whenever our model predicts a TTA below 15 s, an alarm is simulated.

The results from Figure 5 are corroborated, while the average values are presented in Table 3. Importantly, the small error of our model for prediction TTA of 15 s (∼2 s) is well within an acceptable range for practical applications. Taking into account that the detection device is usually deployed 1–1.5 km away from the working site, there is more than half a minute time to take action before the train approaches the working site. Moreover, even in extreme cases where the communication range is very short (e.g., 500 m) and the sensing device cannot be deployed farther away, the additional 10–15 s of TTA prediction significantly contributes to achieving the overall goal.

All the tested ML methods have not missed a single train arrival, i.e., there were no false negatives. This is critical in real-world deployments, as false negatives can lead to catastrophic outcomes, including serious injury and death due to a lack of warning for personnel.

**Figure 6:**
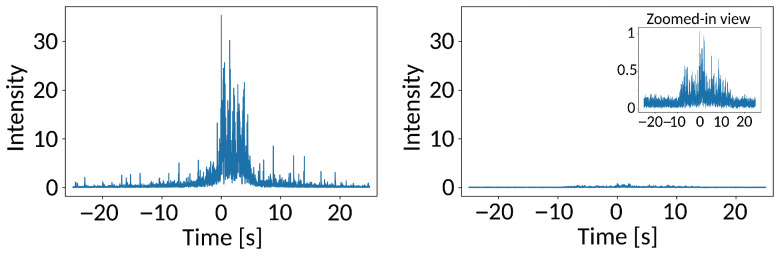
Actual train signal (**left**) and train signal on the parallel track (**right**).

However, there were three false positives, i.e., our model predicted train arrivals where there were none. Further inspection of the signal revealed that the accelerometers also recorded trains passing on the parallel railway tracks in the opposite direction. The intensity of the signal closely resembled the signal of a train arriving in 15 s on “our” tracks, consequently misleading the model. This could be addressed using more sophisticated methods, such as frequency analysis [1] or independent component analysis on top of sensor fusion [36], but the computational requirements are beyond the limits of our edge device setting (see Section 6.2) and require multiple sensors. A more straightforward approach is to slightly increase the threshold that triggers the ML prediction. As shown in Figure 6, the intensity of a signal on the parallel track is much lower than a signal on the actual track. Increasing the threshold will not affect the time prediction result. Currently, this threshold is set manually, but it could also be part of the ML-training process.

## 6. System Implementations and Evaluation in the Field

This section describes the experimental setup as well as the system evaluation. The evaluation is divided in three parts. In the first part, a series of experiments were conducted by feeding data from the real dataset into the microcontroller. This was done by splitting the dataset in small data windows and feeding them one-by-one into the microcontroller. The purpose of the first part was to assess the feasibility of the system to run on resource-constrained devices and whether it can efficiently make decisions on such devices to meet the real-time deadlines set in Section 3. In the second part, the system was deployed on a rail, and its feasibility to detect trains was assessed in real time. The implementation details of a second version of the system are also discussed. Lastly, a power characterization of the system is conducted in the third part.

### 6.1. Implementation

**Figure 7:**
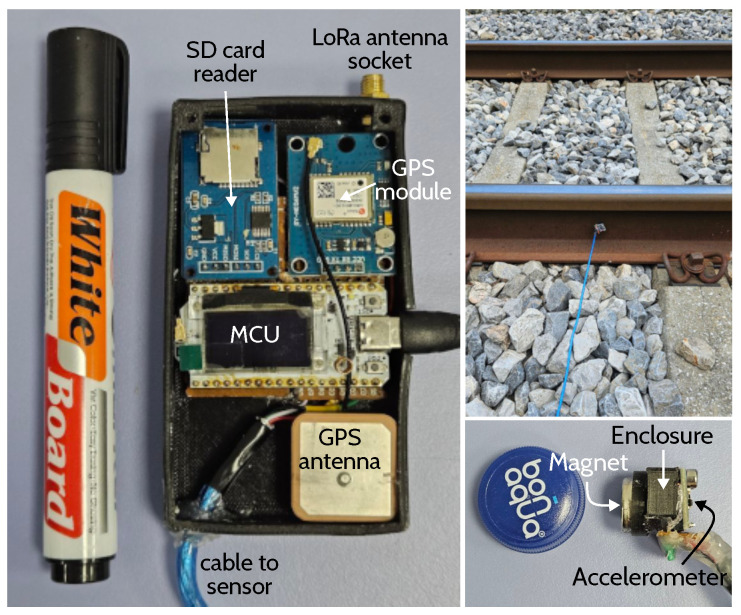
System prototype (version 1.0): components and anchor placement. The SD card module was used for dataset capture; however, in practice, the MCU’s flash memory can be utilized for temporary data storage.

The software was implemented in C++, while the ML model was integrated into the system using the rules extraction method. An ESP32 microcontroller equipped with a LoRa transceiver was selected as a basis for the experimental setup due to its dual-core CPU. The MCU, as well as the rest of the components, is illustrated in Figure 7. The MPU-6050 accelerometer that was used to capture vibrations has a range between −16 and +16g. The recorded measurement rate was one sample every 11 ms, corresponding to a 30 cm train displacement between successive readings at a speed of 100 km/h. While a more frequent data capture is possible, it would increase the amount of data used for ML prediction, thereby extending the inference time. This is further discussed in the next subsection.

Overall, the system is designed to operate without maintenance, aside from occasional battery recharging or replacement. Software updates can be performed by connecting the device to a computer via a USB type-C cable. Over-the-air (OTA) updates are not currently supported; however, since the MCU includes programmable buttons and supports both BLE and Wi-Fi, integrating an existing OTA solution for file transfer would be straightforward.

### 6.2. Ablation: Transformation to Frequency Domain

We conducted a brief experiment on the device to assess the feasibility of signal transformation to the frequency domain. The frequency domain captures slower trends by focusing on lower frequencies, while filtering out noise by disregarding high-frequency intensities. We applied the discrete FFT using the arduinoFFT library (https://github.com/kosme/arduinoFFT (accessed on 29 August 2025)). Since we analyze discrete, non-overlapping windows (see Section 3), transformations over sliding windows, such as the sliding FFT, are not feasible. Thus, FFT needs to be recomputed for each window separately. First, a Hamming windowing function is applied to minimize the spectral leakage. Next, the signal is transformed from the time domain to the frequency domain, and the frequency components are converted to their magnitude representation. Frequency-domain analysis is thus infeasible, as it exceeds our time limit of 1 millisecond. Specifically, benchmarking the discrete FFT computation on 200,000 samples revealed that the FFT-processing time for each data point takes 1753.55±2.53 microseconds. In contrast, computing the intensity for each data point in the window and the median over them takes only 146.45±18.10 microseconds.

In addition, we also retrained the models from Section 5 using the frequency spectrograms as features to predict the TTA. For the signal sampled at 88 Hz and sliding windows of 2 s, this gives 89 features for every training window (23K windows in total). The models trained on the frequency domain did not outperform the models trained on mean intensities, and all achieved a TTA error of over 3 s. This suggests that the filtering of intensities—which is equivalent to removing high frequencies—already produces informative-enough signals for good predictions. In contrast, models learning on frequency spectrograms had to learn this filtering (e.g., by down-weighting the higher frequencies), which increases the difficulty of the task.

Hence, for these two aforementioned reasons, our prediction relies on the time domain, as described in Section 4.1.

### 6.3. Prediction Model and Inference Time

The memory usage of the suggested model primarily consists of storing the two parameters, *a* and *b*. If stored as 32-bit (4-byte) floating-point values, the total memory required is 8 bytes; for 64-bit (8-byte) precision, it requires 16 bytes. This makes the model highly efficient and ideal for low-resource environments, such as the ESP32 or even more resource-constrained MCUs.

Following the extraction and deployment of the model on ESP32, the response time, which includes data collection and preprocessing, median calculation, model inference, and decision transmission time, was recorded throughout the experiments. The results from 2500 samples show that data collection and preparation take 2 s, while the LR model makes its decision in an average of 4.53 microseconds. The median calculation takes approximately 146 microseconds on average. The decision transmission on the second core requires up to 305.15 milliseconds (if LoRa with SF10 is used). As a result, the system’s total response time is less than 3 s; however, this may vary depending on the configuration of the base station (see Section 6.6 for prototype version 2.0).

### 6.4. Long-Range Data Transmission

After the data collection, data processing, and ML inference, the decision about the estimated time arrival of a train is encapsulated into a packet with 16 bytes of payload, optimized for efficient data transfer while including necessary metadata (i.e., device ID, GPS coordinates, timestamp, and decision-related data). Furthermore, all packets include a 4-byte CRC check for data integrity, while the payload is encrypted using AES-128 encryption. One transmission per data buffer window is performed; thus, if a train is identified in multiple consecutive windows (e.g., usually 5–6 in the current design), multiple transmissions with different timestamps will be performed providing redundancy in the system. Moreover, the timestamps from consecutive packets can be used to identify the speed of the train.

The system can be equipped with any radio module to transmit the decision. However, selecting LoRa as the communication method presents several advantages, as also stressed in recent transportation-related studies [37,38,39,40]. Firstly, its long-range capabilities allow for communication over several kilometers, which is beneficial for monitoring and alert systems that cover large geographical areas, such as train tracks or industrial sites. Secondly, LoRa can work in ad-hoc mode without relying on permanent network infrastructures, making it ideal for ad-hoc and remote deployment where no cellular connectivity is available. Finally, LoRa’s low-power nature ensures that devices can operate for extended periods of time without frequent battery replacements, reducing maintenance requirements.

If LoRa is employed as the radio technology, the maximum possible Spreading Factor (a Spreading Factor (SF) is a LoRa modulation parameter that trades data rate for range. It typically ranges from 7 to 12. Higher SF values lead to longer ranges but longer transmission times) that can be used depends on the selected time window. For example, if the time window is 1 s, a SF up to 11 can be used. This allows for a transmission time of 610.3 ms using a typical LoRa channel bandwidth of 125 KHz (the LoRa settings can be adapted to work in regions with limitations in dwell time, such as in the US and Canada. In those regions, SF12 with 500 kHz channel bandwidth, and 25 dBm transmission power could be used). In a series of recent experiments, we examined SF10 and SF11, which achieved a range of 5–7.5 km with Line-of-Sight (LoS) or 1–1.5 km in urban deployments [41]. Since, in the current system, the time window was set to 2 s, SF12 could also be used. However, because the transmission time with this setting is approximately 1.22 s, and the range was already satisfactory with SF10–11, we kept the SF equal to 10 or 11 (TX time = 305.15 − 610.3 ms). Another reason was the radio duty cycle limitation imposed in some regions, which limits the transmission time per hour to 36 s (1%) or 360 s (10%), depending on the selected channel. Since the system may trigger several transmissions per hour, SF10 ensures that the total transmission time is within the duty cycle limits, even with the 1% duty cycle option (i.e., 59–118 transmissions/hour).

### 6.5. System Scalability

The proposed system is well-suited for scalable deployments, even though, in remote railway environments, the infrastructure is sparse and the track layout is relatively simple—typically consisting of one or two parallel rails—without the complexity of large train stations, where many devices could coexist in a small area. In any case, the system’s lightweight communication scheme using LoRa is highly effective, supporting several orthogonal channels for concurrent transmissions and several system coexistence (e.g., up to 8125 KHz channels in the EU433 spectrum or more than 8 in the EU868 spectrum). Moreover, the current TDMA-like schedule (Time Division Multiple Access), which is extensively discussed in our previous work [41], allows for each independent system to operate within four slots, which is translated to up to four detected trains at the same time. Additional slots can be introduced if needed, at the cost of slightly increased latency (e.g., additional 310 ms per slot using SF10), which remains acceptable given the system’s early-warning nature. Moreover, the system can be easily adapted to use a single multi-channel base station capable of supporting up to 8 co-existing systems, equivalent to managing more than 30 sensors simultaneously.

### 6.6. Field Experiments and System Improvements

To test the efficiency of the system in real-time and identify possible design weaknesses, the system was assessed using real field experiments conducted in March and July 2025. In all these experiments, the device was deployed in the middle of the outer rail, between two sleepers of a double-track railway, as depicted in the upper-right part of Figure 7. The first experiment was conducted in March 2025 with cold weather conditions (1 °C and icy), while the second experiment was conducted in July with hot and sunny weather conditions (33 °C in shade). The system was tested on 17 events in total.

In most of the cases, the measurements were taken at a location 4 km beyond the last station in a mostly flat area, where trains typically reach speeds of approximately 100–120 km/h. Across these scenarios, we did not observe any significant fluctuation in noise levels. This suggests that train-induced vibrations are substantially stronger than typical environmental noise. During the experiments, the device was able to capture data on an SD card (this was a necessary step to test other ML approaches in the lab), analyze it in real time, and detect the presence of a train within the time constraints (a minimum of 15 s of notification time). The maximum tested distance between the vibration device and the base station was 1.5 km, which resulted in a minimum notification period of 45 s. At this distance, we recorded an 100% packet success ratio. All 17 passing trains were also detected. Moreover, after analyzing the recorded data in the lab, we observed that LR and XGBoost presented the best performance in terms of RMSE and variance, confirming the initial tests conducted using the captured dataset. The results are summarized in Table 4.

### 6.7. System Improvements

**Figure 8:**
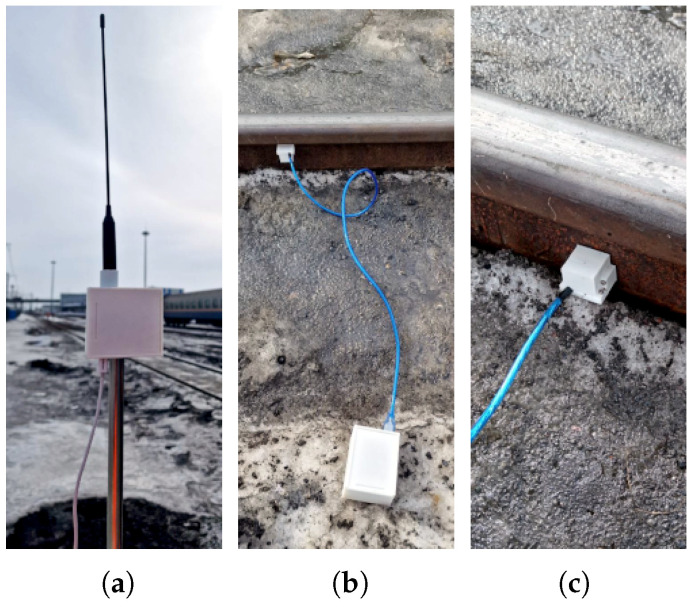
System components of v2.0: (**a**) data-forwarding device; (**b**) vibration sensor device; (**c**) insulated sensor.

A noticeable limitation of the system during the very first set of experiments was the long LoRa antenna cable that was required, considering the fact that the vibration device was placed on the ground (80 cm away from the rails), and the antenna was elevated by at least 1.5 m. This wired design also restricted the possible placement of the antenna post near the tracks, as railroads are typically covered with stones. Thus, a version 2.0 of the system was developed, employing an intermediate communication device near the vibration sensor. The main role of the new device was to forward the data of the vibration sensor device to the base station (via LoRa). The communication between the sensor and the new device was achieved via a 250 Kbps connection-less protocol (ESP-NOW), whose maximum effective range is more than 150 m. A synchronized version of the protocol was also employed to improve energy efficiency in long-term deployments, as described in [42]. This new design provided a much higher flexibility in positioning the antenna (it could even be placed dozens of meters away from the rails) and also provided redundancy by allowing for the connection of multiple sensors at the same time. The new version also employed sensor insulation, as the experiments were conducted with the presence of ice by the rails, as depicted in Figure 8. An additional feature of v2.0 is its notification mechanism, which uses sequential beeping and LED indicators to signal the presence and strength of LoRa connectivity with the base station. This is achieved through occasional beacons transmitted from the base station. The received signal strength (RSS) of these beacons is used to trigger the acoustic and visual indicators.

One drawback of the new version is that the forwarding device must remain continuously powered to receive data, limiting its operational time to approximately 134 h (see next subsection). In contrast, this is not an issue for the first version, as the vibration device can be configured to wake up only when vibrations are detected, extending the battery life to several months. The choice of which system version to use depends on the specific circumstances of the deployment scenario, including factors such as power availability, operational duration requirements, and environmental conditions.

### 6.8. Power Characterization

**Figure 9:**
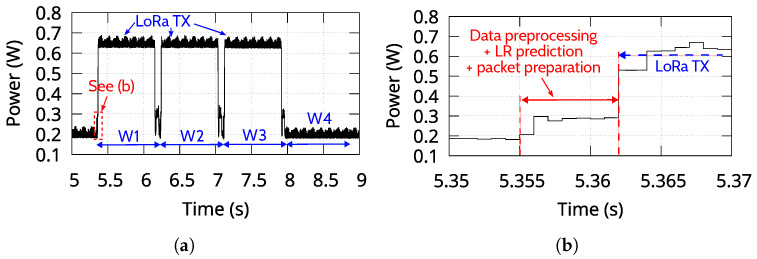
Power characterization of vibration device (v1.0) during simultaneous data collection and LoRa transmission (SF11, 14 dBm). (**a**) Several 1 s windows are shown, with three triggering LR prediction and LoRa transmission. (**b**) Zoomed view of the data analysis and LR prediction period.

A power characterization example of the standard system operation (v1.0) is presented in Figure 9a. To evaluate the timing constraints under strict conditions, we assumed a window size of 1 s and SF11 for LoRa communication. A random train arrival from the dataset is illustrated. The results show that the required power of the data collection running simultaneously with the data processing, the LR prediction, and the LoRa transmission using 14 dBm of transmission power does not exceed 0.7 W. Figure 9b provides a close-up view of data preprocessing, LR prediction, and transmission packet preparation, excluding the power consumption of data collection on Core 0. The entire process completes in under 8 ms.

Assuming that a train passes every 30 min and overall triggers 5 LoRa transmissions, the total energy consumption per train passing is 453.5 Joules. Consequently, a commonly used 10 Ah, 3.8 V battery (i.e., total energy capacity of 38 Wh) can supply power to the system for over 150 h, or more than 6 days. The system expends most of its energy for data capture, with only a small portion used for the ML execution and the LoRa transmissions. However, by setting the microcontroller to wake up only for a triggering accelerometer activity while it is in sleep mode for the rest of the time, the system’s operational lifespan can be extended to several months.

**Figure 10:**
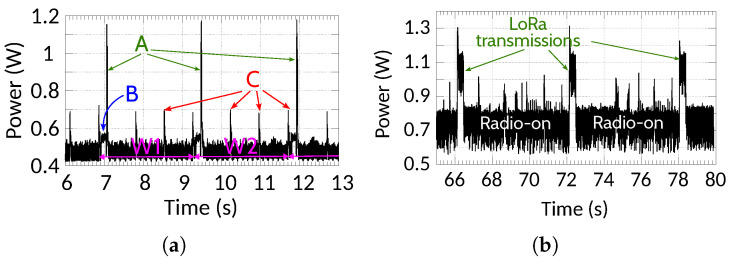
System power characterization (v2.0). (**a**) Vibration device: (A) ESP-NOW transmissions; (B) data preprocessing, LR prediction, and packet preparation; (C) optional SD card writing. (**b**) Data-forwarding device: radio remains on to receive vibration data; LoRa transmissions occur every two beacon periods (6 s).

Finally, the power characterization of the second version of the system is presented in Figure 10. In contrast with v1.0, the vibration device exhibits a much shorter transmission time, reducing the energy consumption. The data-forwarding device has higher power and energy consumption, as it remains *on* to receive data from vibration devices as well as beacons from the base station. To avoid collisions in case of multiple data-forwarding devices, transmissions happen in slots after receiving a beacon. In this particular example, the beacon periodicity is 5.5 s, and the forwarding device uses the second slot to transmit data; thus, there is approximately one transmission every 6 s. The slot selection depends on the direction of the train relevant to the base station. This process is described in detail in our previous work [41], and it is out of the scope of this paper. The average power consumption is 0.744 W, which is translated to 134 h of lifetime using a commonly used 10 Ah battery pack.

### 6.9. Key Outcomes with Respect to System Requirements

Table 5 lists the key outcomes of the system evaluation with respect to the system requirements set in Section 1. The system was evaluated against a set of key functional and non-functional requirements, all of which were successfully met. In terms of core functionality, the system consistently detected the presence of trains in both controlled dataset experiments and real-world field trials. Notification times were also within target, with all trains detected 13 to 16 s before reaching the measurement point. The total system delay to forward alerts to the base station was no more than 2 s, ensuring a worst-case warning window of more than 25 s before a train’s arrival at a hypothetical work site 500 m away, assuming a train speed of 120 km/h.

Power consumption results were also favorable: Version 1.0 of the device achieved several days to weeks of battery life depending on MCU power mode, while Version 2.0—optimized for antenna positioning flexibility—maintained operational life for a few days. Portability was confirmed, with all components weighing under 2 kg and allowing for simple deployment in the field, aside from the optional complexity of placing the LoRa antenna in Version 1.0. Finally, the system remained cost-effective, with the total manufacturing cost of Version 2.0 kept below 500 USD, making it suitable for scalable deployments in resource-constrained environments.

Overall, these outcomes demonstrate that the proposed system not only fulfills its intended safety function but also achieves an effective balance between accuracy, responsiveness, portability, and practicality. The results highlight that a lightweight sensing and communication architecture can provide reliable early-warning capabilities without requiring permanent infrastructure. This makes the approach especially attractive for temporary or remote worksites, where rapid deployment and ease of use are critical.

## 7. Conclusions and Future Work

This study presented a framework for near real-time train detection and notification on resource-constrained IoT devices using accelerometer data and ML. The framework allows for continuous data collection and parallel execution of inference and transmission over LoRa. All tested models achieved 100% accuracy in terms of train detection, but the selected LR model achieved this target with much faster execution times compared to the XGB and LSTM models. Moreover, the evaluation on the rails confirmed that the proposed framework can operate within the desired time constraints and communicate the results within 3 s.

While our dataset reflects real-world conditions, it is limited to a single location. We are planning to expand it across spatial (e.g., urban, rural) and temporal dimensions (e.g., seasons) to better capture and test for local and cyclical patterns. Furthermore, we want to develop a self-learning mechanism to adapt the model to new data in real-time or periodically update itself based on newly collected samples. Moreover, we aim to improve the system’s reachability by integrating Low Earth Orbit (LEO) satellite communications, ensuring connectivity in areas where LoRa may be unreliable, such as hilly terrains. This can be achieved by equipping both the base station and the forwarding device with satellite terminals, allowing them to bypass LoRa when signal quality is poor or connectivity is lost.

## Figures and Tables

**Table 1:** Comparison of literature-proposed and commercial train detection technologies.

Approach/System	Sensing Technology	Infrastructure Requirement	Decision Type	Alert Delay	Alert Range	Power Efficiency
**Scientific Publications and Prototypes**						
Vision-based (Yan et al. [3])	Camera + Convolutional Neural Network (CNN)	No	Binary	<1 s	≤300 m	Medium
Acoustic (Hellman et al. [5])	Microphone array	No	Binary	<1 s	≤500 m	High
Fiber-optic DAS (Du et al. [6])	Distributed acoustic sensing	Yes (cable + terminals)	Estimated TTA (≤15 min)	<0.5 s	≤40 km	Medium
RFID tags (Zhang et al. [7])	Track-mounted tags + readers	Yes (tags + readers)	Binary	<0.5 s	≤100 m	High
LiDAR + GNSS (Dias et al. [8])	LiDAR unit + GNSS	No	Estimated TTA (≤20 s)	<0.5 s	≤500 m	Medium
**Industry Solutions **						
Zöllner MWS [9]	Contact sensors	No	Binary	<2 s	≤3 km	Medium
ZoneGuard [10]	Contactless (infrared etc.)	No	Binary	<2 s	≤3 km	Medium
Protran Detector Kit [11]	Proximity sensor	No	Binary	<2 s	≤1 km	Medium
Minimel Lynx ATWS [12]	Mechanical/inductive sensors	No	Binary	<1 s	≤1 km	Medium
**Proposed System**						
This work	Accelerometer + GPS	No	Estimated TTA (≤15 s)	<2 s	≤5 km	High

**Table 2:** Error of the LR model when predicting TTA of 15 s for each test session.

Window Size (Samples–s)	Session 8 (s)	Session 9 (s)	Session 10 (s)
22–0.25	3.5	7.6	0.2
44–0.5	1.1	4.0	0.5
88–1	1.7	3.5	1.6
**176–2**	**3.2**	**2.9**	**1.0**
264–3	2.7	3.5	2.6

**Table 3:** Detection accuracy, root mean squared error (RMSE) of train time arrival, and false negatives/positives per model.

Approach	Accuracy (%)	RMSE (s)	σ	False Negatives	False Positives
LR	100	2.05	2.48	0	3
XGBoost	100	2.15	2.41	0	3
LSTM	100	2.93	4.02	0	3

**Table 4:** Real field experiments: detection accuracy and root mean squared error (RMSE) of train time arrival per model.

Approach	Accuracy (%)	RMSE (s)	σ
LR	100	3.44	1.29
XGBoost	100	3.18	1.88
LSTM	100	4.14	2.77

**Table 5:** Key evaluation outcomes with respect to system requirements.

Requirement	Achieved?	Details
Train presence detection	Yes	All trains in both the dataset and real-world field tests were successfully detected.
≥15 s notification time	Yes	Trains were detected 13–15 s before reaching the measurement point. The maximum system delay for alert forwarding was 2 s. Even assuming the worst case scenario of a 500 m communication range, the train arrival time to the working site since detection is more than 25 s.
Several hours of lifetime	Yes	Version 1.0 provides several days to weeks of battery life depending on MCU power mode. Version 2.0 achieves up to a few days of operation.
High portability	Yes	All system components are compact, with a total weight under 2 kg. Installation is straightforward, except for potential challenges with positioning the LoRa antenna.
Low cost	Yes	The total manufacturing cost of Version 2.0 does not exceed 500 USD. This cost is derived from the price of the off-the-shelf components used in the system without accounting for additional temperature and water-resistance measures.

## Data Availability

The original data presented in the study are openly available in Zenodo at https://doi.org/10.5281/zenodo.17091186.

## References

[1] Berlin E., Van Laerhoven K. (2012). Trainspotting: Combining Fast Features to Enable Detection on Resource-Constrained Sensing Devices. Proceedings of the 9th International Conference on Networked Sensing (INSS).

[2] Bonde A., Pan S., Mirshekari M., Ruiz C., Noh H.Y., Zhang P. (2020). OAC: Overlapping office activity classification through IoT-sensed structural vibration. Proceedings of the 2020 IEEE/ACM Fifth International Conference on Internet-of-Things Design and Implementation (IoTDI).

[3] Yan F., Gu Y., Sun Y. (2025). Deep Learning-Based Train Obstacle Detection Technology: Application and Testing in Metros. Electronics.

[4] Di Summa M., Griseta M.E., Mosca N., Patruno C., Nitti M., Renò V., Stella E. (2023). A Review on Deep Learning Techniques for Railway Infrastructure Monitoring. IEEE Access.

[5] Hellman A.D., Poirier P.J., John A. (2019). Analysis of Non-Track-Circuit Highway-Rail Grade Crossing Train Detection Technologies.

[6] Du C., Dutta S., Kurup P., Yu T., Wang X. (2020). A Review of Railway Infrastructure Monitoring Using Fiber Optic Sensors. Sens. Actuators A Phys..

[7] Zhang M., Liu Z., Shen C., Wu J., Zhao A. (2022). A Review of Radio Frequency Identification Sensing Systems for Structural Health Monitoring. Materials.

[8] Dias S., Sousa P.J.S.C.P., Nunes J., Afonso F., Viriato N., Tavares P.J., Moreira P.M.G.P. (2025). Deterministic Light Detection and Ranging (LiDAR)-Based Obstacle Detection in Railways Using Data Fusion. Appl. Sci..

[9] Zöllner Signal GmbH Warning Systems for Track Work. https://www.zoellner.de/en/rail/solutions/warning-systems-for-track-work.

[10] RailWorks Corporation ZoneGuard Roadway Worker Protection System. https://www.milleringenuity.com/roadway-worker-protection/.

[11] Protran Technology Portable Train Detector Kit. https://www.protransafety.com/rail/safety/roadway-worker/portable-train-detector-kit.

[12] Schweizer Electronic, AG Minimel Lynx ATWS: Automatic Radio Warning System. https://schweizer-electronic.com/en/warning-systems/temporary-warning-systems/minimel-lynx-atws-automatic-radio-warning-system.

[13] Serna A.A., Yu X., Saniie J. (2024). AI-Based Security Surveillance and Hazard Detection for Train Platform Safety. Proceedings of the 2024 IEEE International Conference on Electro Information Technology (eIT).

[14] Zhang Z., Zaman A., Xu J., Liu X. (2022). Artificial Intelligence-Aided Railroad Trespassing Detection and Data Analytics: Methodology and a Case Study. Accid. Anal. Prev..

[15] Cao Z., Qin Y., Jia L., Xie Z., Gao Y., Wang Y., Li P., Yu Z. (2024). Railway Intrusion Detection Based on Machine Vision: A Survey, Challenges, and Perspectives. IEEE Trans. Intell. Transp. Syst..

[16] Sikora P., Malina L., Kiac M., Martinasek Z., Riha K., Prinosil J., Jirik L., Srivastava G. (2020). Artificial Intelligence-based Surveillance System for Railway Crossing Traffic. IEEE Sens. J..

[17] El-Alami A., Nadir Y., Mansouri K. (2024). A Hybrid Vehicle Tracking System for Low-power Embedded Devices. Proceedings of the 2024 International Conference on Circuit, Systems and Communication (ICCSC).

[18] Alyamkin S., Ardi M., Berg A.C., Brighton A., Chen B., Chen Y., Cheng H.P., Fan Z., Feng C., Fu B. (2019). Low-power Computer Vision: Status, Challenges, and Opportunities. IEEE J. Emerg. Sel. Top. Circuits Syst..

[19] Lin C., Sun G., Wu D., Xie C. (2023). Vehicle Detection and Tracking with Roadside LiDAR Using Improved ResNet18 and the Hungarian Algorithm. Sensors.

[20] Jin X., Yang H., He X., Liu G., Yan Z., Wang Q. (2023). Robust LiDAR-Based Vehicle Detection for On-Road Autonomous Driving. Remote Sens..

[21] Zhangyu W., Guizhen Y., Xinkai W., Haoran L., Da L. (2021). A Camera and LiDAR Data Fusion Method for Railway Object Detection. IEEE Sens. J..

[22] Sato K., Ishida S., Kajimura J., Uchino M., Tagashira S., Fukuda A. Initial Evaluation of Acoustic Train Detection System. Proceedings of the Intelligent Transportation Systems Asia–Pacific Forum Fukuoka.

[23] Rocha G.D., Torres J.C.B., Petraglia M.R., Vorländer M. (2021). Direction of Arrival Estimation of Partial Sound Sources of Vehicles with a Two-Microphone Array. Acta Acust..

[24] Dumont V., Tribaldos V.R., Ajo-Franklin J., Wu K. (2020). Deep Learning for Surface Wave Identification in Distributed Acoustic Sensing Data. Proceedings of the International Conference on Big Data.

[25] Milne D., Masoudi A., Ferro E., Watson G., Le Pen L. (2020). An Analysis of Railway Track Behaviour Based on Distributed Optical Fibre Acoustic Sensing. Mech. Syst. Signal Process..

[26] Sun Y., Cao Y., Liu H., Yang W., Su S. (2023). Condition Monitoring and Fault Diagnosis Strategy of Railway Point Machines Using Vibration Signals. Transp. Saf. Environ..

[27] Meyer A. (2022). Vibration Fault Diagnosis in Wind Turbines Based on Automated Feature Learning. Energies.

[28] Navarro J., Fernández R.R., Aceña V., Fernández-Isabel A., Lancho C., de Diego I.M. (2024). Real-Time Classification of Cattle Behavior Using Wireless Sensor Networks. Internet Things.

[29] Chen J., Wang C., Liu Y. (2024). Vibration Signal Based Abnormal Gait Detection and Recognition. IEEE Access.

[30] Ardiansyah H., Rivai M., Nurabdi L.P.E. (2018). Train Arrival Warning System at Railroad Crossing Using Accelerometer Sensor and Neural Network. AIP Conf. Proc..

[31] Abdel Magid S., Petrini F., Dezfouli B. (2020). Image Classification on IoT Edge Devices: Profiling and Modeling. Clust. Comput..

[32] Elliott D., Otero C.E., Wyatt S., Martino E. (2021). Tiny transformers for environmental sound classification at the edge. arXiv.

[33] Der Yang M., Tseng H.H., Hsu Y.C., Tseng W.C. (2020). Real-Time Crop Classification Using Edge Computing and Deep Learning. Proceedings of the 17th Annual Consumer Communications & Networking Conference (CCNC).

[34] Wang Z., Ling X., Wang L., Zhao Y. (2021). Attenuation Law of Train-Induced Vibration Response of Subgrade in Beijing–Harbin Railway. Shock Vib..

[35] Hsu W.L., Chen W.B., Hsieh M.T. (2024). Comparative Analysis of Methods for Predicting Train-Induced Vibrations. J. Low Freq. Noise, Vib. Act. Control.

[36] Zhang J., Zhang Y., Song B., Zhang Y., Sun J. (2023). Vibration Detection Based on Multi-Sensor Information Fusion for Industrial Internet of Things. Proceedings of the 97th Vehicular Technology Conference (VTC).

[37] Paolini G., Fazzini E., Trovarello S., Amato D., Masotti D., Costanzo A. (2024). An Innovative Multi-Port LoRa-Based Wireless Node for Railway Signaling and Positioning. IEEE J. Radio Freq. Identif..

[38] Suharjono A., Mukhlisin M., Wardihani E.D., Muhlasah Novitasari M., Khusna E.M., Feryando D.A., Adi W.T., Pramono S., Apriantoro R., Mujahidin I. (2023). Performance Evaluation of LoRa 915 MHz for IoT Communication System on Indonesian Railway Tracks with Environmental Factor Propagation Analysis. Proceedings of the 2nd International Conference on Industry 4.0, Artificial Intelligence, and Communications Technology (IAICT).

[39] Torres A.P.A., Silva C.B.D., Filho H.T. (2021). An Experimental Study on the Use of LoRa Technology in Vehicle Communication. IEEE Access.

[40] Li Y., Barthelemy J., Sun S., Perez P., Moran B. (2022). Urban Vehicle Localization in Public LoRaWan Network. IEEE Internet Things J..

[41] Eduard A., Urazayev D., Sabyrbek A., Orel D., Zorbas D. (2024). Ad-hoc Train-Arrival Notification System for Railway Safety in Remote Areas. Internet Things.

[42] Magzym Y., Eduard A., Urazayev D., Fafoutis X., Zorbas D. Synchronized ESP-NOW for Improved Energy Efficiency. Proceedings of the IEEE International Black Sea Conference on Communications and Networking (BlackSeaCom).

